#  Taxonomic changes in palaeotropical Xyleborini (Coleoptera, Curculionidae, Scolytinae)

**DOI:** 10.3897/zookeys.56.520

**Published:** 2010-09-17

**Authors:** Jiri Hulcr

**Affiliations:** 1 Department of Biology, North Carolina State University, Raleigh, NC 27695, USA; 2 Institute of Entomology, CAS, Branisovska 31, 37005 Ceske Budejovice, Czech Republic

**Keywords:** ambrosia beetles, Debus, Microperus, reclassification

## Abstract

Following the recent reclassification of the Palaeotropic xyleborine genera (Hulcr and Cognato in press), additional species are transferred to correct genera or synonymized based on analysis of their morphological characters. The following species are given new combinations: Debus amphicranoides (Hagedorn), **comb. n.**, Debus birmanus (Eggers, 1930), **comb. n.**, Debus dolosus (Blandford, 1896), **comb. n.**, Debus eximius (Schedl, 1970), **comb. n.**, Debus interponens (Schedl, 1954), **comb. n.**, Debus robustipennis (Schedl, 1954), **comb. n.**, Debus spinatus (Eggers, 1923), **comb. n.**, Microperus alpha (Beeson, 1929), **comb. n.**, Microperus corporaali (Eggers), **comb. n.**, Microperus eucalyptica (Schedl, 1938), **comb. n.**, Microperus nugax (Schedl, 1939), **comb. n.**, Pseudowebbia percorthylus (Schedl, 1935), **comb. n.**, Truncaudum circumcinctus (Schedl, 1941), **comb. n.**

The following species are synonymized: Arixyleborus hirtipennis (Eggers), **syn. n.**, with Arixyleborus puberulus (Blandford); Coptoborus palmeri (Hopkins), **syn. n.**, with Debus emarginatus (Eichhoff); Coptoborus terminaliae (Hopkins), **syn. n.**, with Debus emarginatus (Eichhoff); Cyclorhipidion polyodon (Eggers), **syn. n.**, with Truncaudum agnatum (Eggers); Euwallacea artelaevis (Schedl), **syn. n.**, with Planiculus bicolor (Blandford); Xyleborinus perminutissimus (Schedl), **syn. n.**, with Xyleborinus perpusillus (Eggers); Xyleborus exesus Blandford, **syn. n.**, with Debus emarginatus (Eichhoff); Xyleborus fulvulus (Schedl), **syn. n.**, with Microperus corporaali (Eggers); Xyleborus marginicollis (Schedl), **syn. n.**, with Diuncus justus (Schedl); Xyleborus shoreae Stebbing, **syn. n.**, with Debus fallax (Eichhoff).

The following species are given new status: Streptocranus superbus (Schedl, 1951), **restored name**; Webbia divisus Browne, 1972, **restored name**; Webbia penicillatus (Hagedorn, 1910), **restored name**. Genus Taphrodasus Wood (1980) is declared not valid.

## Introduction

Xyleborini are one the most species-rich groups of scolytine beetles, and one which produced many invasive pests. In spite of the economic concern, xyleborine beetles have received comparatively little attention by taxonomists. S.L. Wood (1989) made the first major attempt to organize the many hundreds of described species into a generic classification. This classification was subsequently adopted in the most comprehensive treatise on scolytine taxonomy, the Catalog of Scolytidae and Platypodidae (Wood and Bright 1992, Bright and Skidmore 1997, 2002, also on-line at: http://www.scolytid.msu.edu). This concept was later refined using morphological cladistics (Hulcr et al. 2007a, Hulcr and Cognato 2009), and currently summarized by Alonso-Zarazaga and Lyal (2009). Hulcr and Cognato (in press) provided further rearrangements of Palaearctic and Palaeotropical Xyleborini classification using a combination of morphological and molecular approaches. This work augments the latest classification with a series of transfers and synonyms. Majority of the species treated here occur in SE Asia or Melanesia.

### List of abbreviations

BMNHNatural History Museum, London

FRIForestry Research Institute, Dehra Dun, India

MCgMuseo Civico Genova, Genova

MNBMuseum fur Naturkunde der Humboldt University, Berlin, Germany

MSUCMichigan State University Arthropod Collection, East Lansing, MI, USA

NHMWNatürhistorisches Museum, Wien, Austria

RABRoger A. Beaver’s private collection, Chiang Mai, Thailand

SMTDStaatliches Museum fur Tierkunde, Dresden, Germany

UCDBohart Museum, University of California-Davis, CA, USA

USNMUnited States National Museum, Smithsonian Institution, Washington, D.C., USA.

## Taxonomic treatment

### Arixyleborus puberulus

(Blandford)

Xyleborus puberulus  (Blandford, 1896)
Xyleborus hirtipennis  Eggers, 1940, **syn. n.**
Arixyleborus hirtipennis  (Eggers): Browne 1955, **syn. n.**

#### Specimens examined.

Indonesia, Java (Xyleborus hirtipennis, lectotype, USNM); Sarawak, Malaysia (Xyleborus puberulus, holotype, BMNH).

#### Comments.

Lectotype of Arixyleborus hirtipennis bears all essential features of Arixyleborus puberulus, only the declivital rugosities more organized into rows, shining area of elytra smaller, less clearly distinguished from rugose area. These are exceptionally plastic in Arixyleborus puberulus, Arixyleborus hirtipennis represents small deviation in the large range of variation of declivital surface in Arixyleborus puberulus.

### Diuncus justus

(Schedl)

Xyleborus justus  Schedl, 1931
Diucus justus  (Schedl): Hulcr and Cognato 2009
Xyleborus marginicollis  (Schedl, 1936b), **syn. n.**

#### Specimens examined.

Indonesia, Java, Buitenzorg (Diuncus justus, holotype, NHMW); Philippines, Luzon, (Xyleborus marginicollis, holotype, NHMW).

#### Length.

1.5 mm.

#### Comments.

Type specimen of Xyleborus marginicollis Schedl represents one end of a continuum of variation in Diuncus justus: short (1.5 mm) but robust (most representatives of Diuncus justus slightly longer and more slender). Diagnostic characters identical: surface of declivity devoid of vestiture, no elytral denticles, smooth impression across interstriae 2 and 3 (very shallow).

### Debus amphicranoides

(Hagedorn) comb. n.

Xyleborus amphicranoides  Hagedorn, 1908

#### Specimens examined.

Malaysia, Sabah, Danum Valley (2, R.A. Beaver det., MSUC); Sumatra (USNM).

#### Comments.

Prolonged large representative of Debus. Elytral declivity deeply excavated, edge of declivity with two pairs of long teeth, but only few tubercles. Declivital surface smooth.

Debus amphicranoides (Hagedorn) possibly senior synonym of the following (NHMW): Debus birmanus (Eggers), Debus cyclopus (Schedl), Debus interponens (Schedl), Debus robustipennins (Schedl). Debus birmanus identical except slightly larger, with slightly longer declivital posterolateral processes, much smaller upper tooth on declivity. Debus interponens similar except lacks constricted declivity and has shorter posterolateral declivital processes. Schedl (1954) considered Debus (as Xyleborus) interponens possible altitudinal variation of Xyleborus robustipennins; the two essentially identical, originated from different elevations. Debus cyclopus similar except narrower elytral apical emargination. Debus robustipennis larger. Schedl (1954) mentioned that it only differed from Debus amphicranoides in minor differences in declivital teeth shape.

### Debus birmanus

(Eggers) comb. n.

Xyleborus birmanus  Eggers, 1930

#### Specimens examined.

Malaysia, Burma (2 indiv., BMNH).

#### Comments.

Very similar to Debus amphicranoides, possibly a synonym. Holotype at FRI not available.

### Debus dolosus

(Blandford) comb. n.

Xyleborus dolosus  Blandford, 1896

#### Specimens examined.

Malaysia, Sarawak (holotype, BMNH); Malaysia, Sabah, Danum Valley (9 indiv., Hulcr det., MSUC.).

#### Diagnosis.

Elytral declivity slightly with much higher number of declivital tubercles than other Debus. Declivity flat, not excavated, not emarginate at apex. Depth of emargination varies. Similar to Debus pumilus, but uniformly brown, with more and larger tubercles on the declivity. Significant intraspecific size variation.

#### Comments.

Elytral declivity superficially different from other Debus spp, but its structure homologous. Few small or large tubercles in the interstriae 1 (usually 3 pairs), displaced by broadened interstriae 1 and positioned on first striae or on interstriae 2. Strial punctures greatly reduced on declivity, difficult to follow as interstria 1 broad, displacing other striae. No tubercles originating on second striae. Smaller tubercles on striae 3 and beyond, creating tuberculated area surrounding declivity. Other characters shared with Debus spp.: extended pronotal disc, triangular protibiae with large and long but sparse denticles (<7), inflated prosternal posterocoxal process, antennal club shape.

Xyleborus persimilis (Eggers) and Debus dolosus (Blandford) probably synonyms. Xyleborus persimilis (lectotype, USNM) with slightly broader, more excavated declivity. Browne (1961) suggested that Xyleborus subdolosus is only a local variety of Debus dolosus.

### Debus eximius

(Schedl) comb. n.

Xyleborus eximius  Schedl, 1970

#### Specimens examined.

Indonesia, Kalimantan (2, holotype & allotype, NSMT); Indonesia, Kalimantan (2 paratypes, NHMW).

#### Comments.

Elytral apex not emarginate, but all other diagnostic characters of Debus present: elongated pronotal disc, broad antennal club type 2, triangular protibiae, flat elytral declivity with tubercles on elevated lateral sulcus (appears as if formed by interstriae 2 through 4).

### Debus fallax

(Eichhoff)

Xyleborus fallax  Eichhoff, 1878
Debus fallax  (Eichhoff): Hulcr and Cognato in press
Xyleborus shoreae  Stebbing, 1909, **syn. n.** (complete taxonomic history in Wood and Bright 1992)

#### Specimens examined.

Xyleborus shoreae: India, Kumaon, (2), Beeson det., BMNH); Malaysia, Kedah, (two labels: Xyleborus shoreae, Browne det., Xyleborus fallax, Schedl det., BMNH); Thailand, Pong Yaeng N. P., (Beaver det.); Borneo (Schedl det., BMNH); Debus fallax: Malaysia, Sabah, Danum Valley (Hulcr det.); Malaysia, Sabah, Danum Valley (51, Hulcr det.); New Guinea, Morobe Province, Bulolo (Jordal det.); New Guinea (BBM, 5 indiv.); Sulawesi (Browne det., BMNH); Thailand, Pong Yaeng N. P. (2, Hulcr det.); PNG, Madang Prov. (36), Oro Prov. (66), West Sepik (123) (Hulcr coll, det.); Philippines, Luzon, Mt. Makiling (Xyleborus amphicranulus Egg. holotype, Xyleborus fallax syn., SMTD).

#### Comments.

Holotype of Xyleborus shoreae in FRI, inaccessible, non-type specimens identified by several authorities available. Location of Xyleborus fallax holotype unclear. Wood and Bright (1992) indicated IRSNB as holotype depository, however museum personell reports that holotype has never been deposited there. Xyleborus shoreae is a variant of Debus fallax (Eichhoff), declivital emargination shallower than in most Debus fallax. All other characters identical to Debus fallax: color uniformly brown to bicolored (light brown to orange pronotum), elytral denticles all small except the denticle in the middle of declivital face, which is slightly longer than others; declivity surface shining, most specimens with remnants of strial punctures, size 2.6 - 3.0 mm. Declivity emargination depth intermediate between Debus fallax and Debus emarginatus, most other characters (size, coloration, flat posterolateral processes) shared with Debus fallax. Maiti and Saha (2004) had access to holotype, redescription and illustration fits Debus fallax. Stebbing not consistent in distinguishing Xyleborus shoreae from Xyleborus fallax, assigned similar specimens to either species (Maiti and Saha 2004).

#### Biology and distribution:

Reported from India and Thailand, and by Browne (1983) as imported from PNG to Japan. Despite the name “shoreae”, the species is a broad generalist (Wood and Bright 1992).

### Debus interponens

(Schedl) comb. n.

Xyleborus interponens  Schedl, 1954

#### Specimens examined.

Malaysia, Sarawak, Mt. Penrissen, 4500 ft. (lectotype, NHMW).

#### Comments.

All diagnostic features of genus Debus present, including antennal club form, prolonged pronotum, emarginate declivity. Similar to Debus amphicranoides (Hagedorn), but with less constricted declivity and longer posterolateral declivital processes. Schedl (1954) considered Debus interponens altitudinal variant of Debus robustipennins, the two are allegedly identical, only differing by their origins from different elevations.

### Debus robustipennis

(Schedl) comb. n.

Xyleborus robustipennis  Schedl, 1954

#### Specimens examined.

Indonesia, Borneo (lectotype, NHMW).

#### Comments.

All diagnostic features of Debus present, including antennal club form, prolonged pronotum, emarginate declivity.

Lectotype of Xyleborus robustipennis Schedl very similar to non-type specimens of Debus amphicranoides (Hagedorn) in USNM, only slightly larger. Schedl (1954) indicated that Xyleborus robustipennis differs from Xyleborus amphicranoides very little, merely by shallower and wider declivital emargination, having the lateral declivital costa between teeth 1 and 2 more elevated, and lateral declivital process shorter. Type of Debus amphicranoides not available, thus synonymy could not be confirmed.

### Debus spinatus

(Eggers) comb. n.

Xyleborus spinatus  Eggers, 1923

#### Specimens examined.

Malaysia (BMNH); Malaysia, Sabah, Danum Valley (3, Hulcr det.).

#### Diagnosis.

An “elegant” form of Debus fallax. Longer, smooth declivity, shallowly emarginate, no tubercles or granules on declivital sides except two pairs of slender teeth, one long, one short. Declivity shagreen when dry.

### Debus emarginatus

(Eichhoff)

Xyleborus emarginatus  Eichhoff, 1878
Debus emarginatus  (Eichhoff): Hulcr and Cognato in press
Xyleborus terminaliea  Hopkins, 1915, **syn. n.**
Coptoborus terminaliae  (Hopkins) Wood and Bright 1992, **syn. n.**
Xyleborus exesus  Blandford, 1894, **syn. n.**
Xyleborus palmeri  Hopkins, 1915, **syn. n.**
Coptoborus palmeri  (Hopkins): Wood and Bright 1992, **syn. n.** (complete taxonomic history in Wood and Bright 1992)

#### Specimens examined.

Xyleborus terminaliae: Philippines, Pagbilao (holotype, USNM). Xyleborus exesus: Japan, (holotype, BMNH). Debus emarginatus: Indonesia, Sumatra, Bandar Baroe (homotype, compared to type by Eggers, NHMW); Indonesia, Java, Bandjar (homotype, compared to type by Eggers, NHMW, 2 indiv.); Philippines, Laguna, Pangil (homotype, NHMW); Malaysia, Sabah, Danum Valley (17 indiv., Hulcr det., MSUC); New Guinea (BBM, 20 indiv.); New Guinea, Ambunti (4, BBM); New Guinea (FICB); New Guinea, Gulf Province, Ivimka (UCD); Thailand, Pong Yaeng N. P. (Hulcr det., MSUC); PNG, Madang Prov. (79, Hulcr coll.).

#### Comments.

Holotypes of Xyleborus exesus Blandford, Xyleborus palmeri Hopkins, and Xyleborus terminaliae Hopkins share all diagnostic characters with homotype and large series of non-types of Debus emarginatus (Eichhoff). Xyleborus exesus: declivity with slightly less steep slope, less pronounced lateral tubercles (granules), dominant tubercle in middle of lateral sulcus slightly longer. Schedl (1973e) suggested synonymy of non-New Guinean Xyleborus emarginatus Schedl with Xyleborus exesus Blandford, based on shared shallow declivital emargination. Holotype of Xyleborus exesus damaged, missing elytron, fits range of Debus emarginatus variation. Xyleborus palmeri Hopkins is larger variant of typical Debus emarginatus.

### Microperus alpha

(Beeson) comb. n.

Xyleborus alpha  Beeson, 1929
Coptodryas alpha  (Beeson): Wood and Bright 1992

#### Specimens examined.

India, Sunderbans Div. (holotype, BMNH).

#### Comments.

All diagnostic features of Microperus present: small size, elytral punctures aligned in striae, and prolonged body shape (Hulcr and Cognato in press). Similar to Microperus pometianus, but slightly longer, with distinctly elevated and long declivital costa.

### Microperus corporaali

(Eggers) comb. n.

Xyleborus corporaali  (Eggers, 1923)
Coptodryas corporaali  (Eggers): Wood and Bright 1992
Xyleborus fulvulus  (Schedl, 1942b), **syn. n.**
Xyleborus fulvus  (Schedl, 1939): Xyleborus fulvulus (Schedl, 1942), preoccupied (Xyleborus fulvusMurayama 1936), **syn. n.**

#### Specimens examined.

Microperus corporaali: Indonesia, Kotangan an der Ostkusgte (lectotype, USNM); Xyleborus fulvulus: Indonesia, Sumatra (paratype, USNM).

#### Comments.

Xyleborus fulvulus identical to Microperus corporaali (identical antennae, body shape, declivital surface and shape, posterolateral declivital costa, declivital vestiture as one row of erect setae per intrestria, backward-bent setae in strial punctures). Paratype not mentioned by Anderson and Anderson (1971).

### Microperus eucalyptica

(Schedl) comb. n.

Xyleborus eucalyptica  Schedl, 1938
Coptodryas eucalyptica  (Schedl): Wood and Bright 1992

#### Specimens examined.

Australia, Queensland, Geagana (lectotype, NHMW).

#### Comments.

All diagnostic features of Microperus present (elytral mycangia, absence of scutellum, small size, prolonged body shape, abundant vestiture). Similar to Microperus intermedius, but substantially longer, elytra often bicolored, usually without convexity on elytral disc.

### Microperus nugax

(Schedl) comb. n.

Xyleborus nugax  Schedl, 1939
Coptodryas nugax  (Schedl): Wood and Bright 1992

#### Specimens examined.

Malaysia, Selangor (lectotype, BMNH); Malaysia, Selangor (Schedl det., BMNH); Malaysia, Sabah, Danum Valley, (13, Hulcr det., MSUC).

#### Diagnosis.

Very similar to Microperus diversicolor (e.g., antennal club type 3), except pronotum bright yellow with brown patch, elytra black, declivity commencing closer to elytral base, declivital interstriae covered with many small sharp hooks (similar as in Microperus parva, but larger). Characteristic elytral disc: anterior portion inflated, convex, boundary between elytral disc and declivity slightly concave, impressed.

#### Comments.

Schedl (1979) designated lectotype in NHMW, another unspecified “type” resides in BMNH. Possibly synonymous with Coptodryas undulata (Sampson) (as Xyleborus leprosulus Schedl, syn. Wood 1989) (Schedl 1939).

#### Biology.

Creates irregularly branching galleries with transverse brood chambers (Beaver and Browne 1978).

### Planiculus bicolor

(Blandford)

Xyleborus bicolor  Blandford, 1894
Euwallacea bicolor  (Blandford): Wood and Bright 1992
Planiculus bicolor  (Blandford): Hulcr and Cognato 2010
Xyleborus artelaevis  (Schedl, 1942a), syn. n.
Euwallacea artelaevis  (Schedl): Beaver 1998, syn. n.
Xyleborus rameus  Schedl, 1940
Xyleborus bicolor  (Schedl): Kalshoven 1959

#### Specimens examined.

Xyleborus artelaevis: Malaysia, Perak, (holotype, NHMW); New Guinea, Gulf Province, Ivimka, (R.A. Beaver det., UCD); Indonesia, Sulawesi (R. A. Beaver det., BMNH). Planiculus bicolor: Nagasaki, Japan (syntype, BMNH); Fiji, Namosi (Xyleborus rameus (syn. Planiculus bicolor) Schedl det., BMNH).

#### Comments.

Holotype of Xyleborus artelaevis virtually identical to Planiculus bicolor (Blandford), except first segment of antennal club more convex. All other characters identical, including uniform granules in declivital interstriae 1, 2, and 3 (same size granules in interstriae 1–3 characteristic for Planiculus bicolor). Xyleborus artelaevis holotype deteriorated, missing or damaged body parts including antenne.

### Pseudowebbia percorthylus

(Schedl) comb. n.

Xyleborus percorthylus  Schedl, 1935
Taphrodasus percorthylus  (Schedl): Wood 1980

#### Specimens examined.

Malaysia, Peninsula (holotype, NHMW).

#### Comments.

Diagnostic characters of Pseudowebbia: regular type of pronotum (not extremely prolonged and flat as in Webbia), circular antennal club (not broadened), triangular to broadly rounded protibia (not thin and sickle-like as in Webbia). Elytral declivity deeply excavated, surrounded by highly elevated circumdeclivital costa with no teeth.

Type species of Taphrodasus Wood, 1980. Morphological limits of Taphrodasus never specified. Characters listed by Wood (1980) are either autapomorphic to Taphrodasus percorthylus, or present in other genera, mostly Webbia. Taphrodasus not a valid genus, see below.

### Streptocranus superbus

(Schedl) stat. n.: restored name

Xyleborus superbus  Schedl, 1951
Coptoborus superbus  (Schedl): Wood and Bright 1992
Xyleborus superbulus  Schedl, 1958a, **unnecessary replacement name**
Coptoborus superbulus  (Schedl, 1958a): Wood and Bright 1992, **unnecessary replacement name**

#### Specimens examined.

Indonesia, Java, Buitenzorg (holotype, NHMW).

#### Comments.

Xyleborus superbus Schedl (1951) preoccupied by Xyleborus superbus Schedl (1942c). Renamed Xyleborus superbulus (Schedl, 1958a). Xyleborus superbus Schedl (1942c) later placed in Coptoborus (Wood and Bright 1992). Replacement name unnecessary, original name Streptocranus superbus (Schedl, 1951) restored.

### Genus Taphrodasus

Wood stat. n.: invalid genus

Taphrodasus Wood (1980), monotypic, type species Taphrodasus percorthylus (Schedl, 1935): Wood 1980. Later included in Taphrodasus: Webbia divisusBrowne (1972), Xyleborus penicillatusHagedorn (1910), Xyleborus cuspidus Schedl (1975). Taphrodasus percorthylus transferred to Pseudowebbia (Hulcr and Cognato, this volume); Taphrodasus divisus and Taphrodasus penicillatus restored in Webbia (Hulcr and Cognato, this volume), Taphrodasus cuspidus not related to any of the other three species (Hulcr and Cognato, in prep.).

### Truncaudum agnatum

(Eggers)

Xyleborus agnatus  Eggers, 1923
Cyclorhipidion agnatum  (Eggers): Wood and Bright 1992
Truncaudum agnatum  (Eggers): Hulcr and Cognato 2010
Xyleborus polyodon  (Eggers, 1923), **syn. n.**
Cyclorhipidion polyodon  (Eggers, 1923): Wood and Bright 1992, **syn. n.** (complete taxonomic history in Wood and Bright 1992)

#### Specimens examined.

Truncaudum agnatum: New Guinea, Hatam (cotype, MCG). Xyleborus polyodon: Philippines, Luzon, Mt. Makiling; (unspecified “type”, SMTD).

#### Comments.

Type of Xyleborus polyodon similar to Truncaudum agnatum, except tubercles on and around declivity larger, pointed. Tubercles in homologous position. Eggers’s unspecified “type” in SMTD from the same collection series as lectotype at USNM (Anderson & Andreson, 1971).

### Truncaudum circumcinctus

(Schedl) comb. n.

Premnobius circumcinctus  Schedl, 1941
Premnobius circumcinctus  (Schedl): Wood and Bright 1992
Xyleborus circumcinctus  (Schedl): Schedl 1962b

#### Specimens examined.

Uganda (Premnobius circumcinctus, holotype, NHMW).

#### Comments.

The only known African Truncaudum. Truncaudum synapomorphies: impressed submentum, antennal club type 1, curved outer edge of protibiae, complete denticulated circumdeclivital costa. Very similar to Truncaudum impexus [(Schedl): Hulcr and Cognato (in press)], except declivity slightly convex (mostly flat in Truncaudum spp.), antenna type 1, several adjacent denticles on each stria on the upper edge of circumdeclivital costa (mostly a single flat tubercle in Truncaudum spp.). Otherwise remarkably similar to Asian relatives. Length: 2.8 mm.

Described as Premnobius by Schedl (1941), later treated as Xyleborus (Schedl, 1962b), but combination never offically published.

### Webbia divisus

Browne stat. n.: restored name

Webbia divisus Browne (1972)
Taphrodasus divisus  (Browne): Wood and Bright 1992

#### Specimens examined.

Malaysia, Perak (holotype, BMNH).

#### Comments.

Transferred to Taphrodasus (Wood & Bright, 1992) without discussion of characters. Webbia synapomorphies: dorsal aspect of pronotum long and quadrangular, pronotal disc long and flat, frontal slope of pronotum short, scutellum suppressed, costate and setose elytral bases. Differs from most Webbia spp. by densely pubescent and excavated declivity and elongated body shape. Length: 2.4 mm. Characters shared with Pseudowebbia percorthylus [(Schedl, 1935): Hulcr and Cognato, this volume] (type species of Taphrodasus) limited to excavated declivity with dense setae, genus-level characters different.

### Webbia penicillatus

(Hagedorn) stat. n.: restored name

Xyleborus penicillatus Hagedorn 1910
Prowebbia penicillatus   (Hagedorn): Browne 1963
Webbia penicillatus   (Hagedorn): Bright 2000
Taphrodasus penicillatus   (Hagedorn): Wood and Bright 1992

#### Specimens examined.

Malaysia, N.S. Triang (homotype, NHMW); Malaysia, Perak (BMNH); Malaysia, Borneo (BMNH).

#### Comments.

Type in Hamburg museum lost (Wood and Bright 1992).Most features characteristic of Webbia (elongated pronotum, suppressed scutellum), unrelated to type species of Taphrodasus: Pseudowebbia percorthylus ((Schedl, 1935): Hulcr & Cognato, this volume). Similar to Webbia divisus, except declivity with long, dense, erect setae, not scales.

### Xyleborinus perpusillus

(Eggers)

Xyleborus perpusillus  Eggers, 1927
Xyleborinus perpusillus  (Eggers): Wood & Bright, 1992
Xyleborinus perminutissimus   (Schedl, 1934) **syn. n.**

#### Specimens examined.

Xyleborinus perminutissimus: Indonesia, Java, Mt. Gede (lectotype, NHMW). Xyleborinus perpusillus: Indonesia, Sumatra (holotype, USNM); Malaysia, Sarawak, Gunung Buda (R.A. Beaver det., MSUC); Malaysia, Sabah, Danum Vallery (29 indiv., Hulcr coll.); New Guinea, Oro Province, Kanga (5 indiv, Hulcr coll.).

#### Comments.

Lectotype of Xyleborinus perminutissimus (Schedl, 1934d) virtually identical to holotype of Xyleborinus perpusillus (Eggers). Slightly smaller tubercles in some declivital interstriae, but pattern identical: tubercles missing from interstriae 2.

## Supplementary Material

XML Treatment for Arixyleborus puberulus

XML Treatment for Diuncus justus

XML Treatment for Debus amphicranoides

XML Treatment for Debus birmanus

XML Treatment for Debus dolosus

XML Treatment for Debus eximius

XML Treatment for Debus fallax

XML Treatment for Debus interponens

XML Treatment for Debus robustipennis

XML Treatment for Debus spinatus

XML Treatment for Debus emarginatus

XML Treatment for Microperus alpha

XML Treatment for Microperus corporaali

XML Treatment for Microperus eucalyptica

XML Treatment for Microperus nugax

XML Treatment for Planiculus bicolor

XML Treatment for Pseudowebbia percorthylus

XML Treatment for Streptocranus superbus

XML Treatment for Taphrodasus

XML Treatment for Truncaudum agnatum

XML Treatment for Truncaudum circumcinctus

XML Treatment for Webbia divisus

XML Treatment for Webbia penicillatus

XML Treatment for Xyleborinus perpusillus
